# Knowledge and acceptability of *Chlamydia trachomatis* screening among pregnant women and their partners; a cross-sectional study

**DOI:** 10.1186/1471-2458-14-704

**Published:** 2014-07-09

**Authors:** Monique TR Pereboom, Evelien R Spelten, Judith Manniën, G Ingrid JG Rours, Servaas A Morré, François G Schellevis, Eileen K Hutton

**Affiliations:** 1 Department of Midwifery Science, AVAG and the EMGO + Institute for Health and Care Research, VU University Medical Center, P.O. Box 7057, (Room D4.40), Amsterdam, 1007 MB, The Netherlands; 2 Department of Infectious Disease Epidemiology, Imperial College School of Public Health, Imperial College London, Norfolk Place, London, W2 1PG, UK; 3 Department of Medical Microbiology and Infectious Diseases, Erasmus Medical Centre, Rotterdam, The Netherlands; 4 Department of Paediatrics, Erasmus Medical Centre, Rotterdam, The Netherlands; 5 Laboratory of Immunogenetics, Department of Medical Microbiology and Infection Control, VU University medical center, Amsterdam, The Netherlands; 6 Institute for Public Health Genomics, Department of Genetics and cell Biology, Research School GROW, Maastricht University, Maastricht, The Netherlands; 7 Netherlands Institute for Health Services Research (NIVEL), P.O. Box 1568, Utrecht, 3500, BN, The Netherlands; 8 Department of General Practice and Elderly Care Medicine/EMGO+, Institute for Health and Care Research, VU University Medical Center, P.O. Box 7057 (Room D5.38), Amsterdam, 1007 MB, The Netherlands; 9 Faculty of Health Sciences, McMaster University, Michael G. DeGroote Centre for Learning (Room 2210), 1280 Main St. W, Hamilton, Ontario L8S 4 K1, Canada

**Keywords:** *Chlamydia trachomatis*, Prenatal care, Health knowledge, Attitudes, Experiences, Pregnancy

## Abstract

**Background:**

*Chlamydia trachomatis* infections in pregnancy can cause maternal disease, adverse pregnancy outcomes and neonatal disease, which is why chlamydia screening during pregnancy has been advocated. The effectiveness of a screening program depends on the knowledge of health care professionals, women and partners and the acceptability for screening of the target population. We assessed the knowledge of chlamydia infection among pregnant women and their partners in the Netherlands, their attitudes towards testing, and their experiences of being offered a chlamydia test. In addition, we evaluated the association between participants’ background characteristics and knowledge of chlamydia.

**Methods:**

Pregnant women aged ≤ 30 years and their partners (regardless of their age) attending one of the participating primary midwifery care practices in the Netherlands were invited to participate. All participants completed a questionnaire, pregnant women provided a vaginal swab and partners provided a urine sample to test for *C. trachomatis*.

**Results:**

In total, 383 pregnant women and 282 partners participated in the study of whom 1.9% women and 2.6% partners tested chlamydia positive. Participants had high levels of awareness (92.8%) of chlamydial infection. They were knowledgeable about the risk of chlamydia infection; median knowledge score was 9.0 out of 12.0. Lower knowledge scores were found among partners (p-value <0.001), younger aged (p-value 0.02), non-western origin (p-value <0.001), low educational level (p-value <0.001), and no history of sexually transmitted infections (p-value <0.001). In total, 78% of respondents indicated that when pregnant women are tested for chlamydia, their partners should also be tested; 54% believed that all women should routinely be tested. Pregnant women more often indicated than partners that testing partners for chlamydial infection was not necessary (p-value <0.001). The majority of pregnant women (56.2%) and partners (59.2%) felt satisfied by being offered the test during antenatal care.

**Conclusion:**

Pregnant women and their partners were knowledgeable about chlamydial infection, found testing, both pregnant women and their partners, for chlamydia acceptable and not stigmatizing.

## Background

*Chlamydia trachomatis* infection in pregnancy can cause maternal disease, adverse pregnancy outcomes, and neonatal disease [1-5]. High chlamydia prevalence rates have been described among pregnant women varying from 3.2% to 5.9%, with even higher rates among pregnant teenagers (14%) [1,6-9]. In general, approximately 80% of infected women and 50% of infected men are asymptomatic or minimally symptomatic. Hence, screening is the only means to effectively identify infections [2].

In the Netherlands pregnant women are routinely tested for HIV, syphilis and hepatitis B, but not for chlamydia [2,10]. International guidelines recommend universal chlamydia screening during antenatal care or screening of pregnant women less than 25 years of age [11-15]. Studies suggest using either of these approaches for routine chlamydia screening is cost effective [6,16]. The Dutch Health Council has no recommendation specific for pregnant women, but recommends in general that health care professionals should actively screen for chlamydial infections in people who are at higher risk [17]; the latter being young age, Surinamese or Antillean ethnicity, attending clinics for STIs, having multiple sexual partners, and other risk factors in combination with sexual behaviour or symptoms, partners of chlamydia positive persons, and mothers of chlamydia positive new-borns [17]. However, in a previous study we showed that the decision for Dutch midwives to offer chlamydia testing to pregnant women is based on symptoms rather than on risk factors [18]. Hence, many chlamydial infections will remain undetected.

A key factor for the effectiveness of an antenatal chlamydia screening program is that women and their partners have knowledge about the infection and that they accept screening [19]. Only few studied the knowledge of chlamydia, attitudes towards chlamydia infection screening and experiences of being offered a chlamydia test among pregnant and non-pregnant women, and their partners [20,21].

The aim of this study was to assess the knowledge of chlamydia infection among pregnant women and their partners in the Netherlands and to determine associations between pregnant women and their partner’s demographic characteristics and their knowledge on chlamydia infections. In addition, we assessed their attitudes towards antenatal chlamydia testing and experiences of being offered a test in antenatal care.

## Methods

This study is part of a national cross-sectional study about the prevalence and risk factors for chlamydia infection in pregnant women and their partners. Primary care midwifery practices were invited using a sampling method based on the location of the practices in the Netherlands. In total, twenty-two primary midwifery care practices participated. Pregnant women were eligible for participation if they consulted a midwife in one of the participating practices between May 2012 and December 2013, were pregnant at the time of enrolment, had reached the legal age of consent of 18 years, were younger than 31 years of age, and were able to understand Dutch. Partners of women were eligible to participate if they were present at the time their pregnant partner was included, and were able to understand Dutch. Because chlamydia is more prevalent among younger people, we decided to include only pregnant women younger than 31 years. There was no age limit for the partners.

The Medical Ethics Committee of the VU University Medical Center Amsterdam, the Netherlands, approved the study.

### Data collection

The midwife or practice assistant informed pregnant women and their partners about the study and invited them to participate. Eligible pregnant women and partners signed an informed consent form. They were asked to fill in a questionnaire, which contained 37 questions. In addition they were asked to provide a self-collected sample (e.g. a vaginal swab for women and urine specimen for partners), which was sent to the laboratory for *C. trachomatis* testing.

The questionnaire was developed to obtain data on demographic characteristics, knowledge of the infection, and attitudes towards testing for chlamydia in antenatal care. Questions were based on previous studies and the literature [8,19,22-24]. Questionnaires were provided with a prepaid return envelope. The informed consent forms and questionnaires were provided with an unique anonymized identification number. We conducted a small pilot study and confirmed the acceptance of this relatively personal questionnaire among women and their partners. Demographic characteristics and risk factors were age at the time of enrolment, highest achieved level of education, ethnic origin, urbanisation level, marital status (no partner/having a partner, but living alone/married or living with a partner), gravidity (primigravidae versus multigravidae), planned pregnancy (yes/no) and history of sexually transmitted infections (STI) (yes/no/never been tested). For the analyses we categorized some of the demographic characteristics of the participants. Age was defined as the age at enrolment and categorized into three groups for women: ≤20 years, 21–25 years, 26–30 years. For partners we used the same age groups as well as an additional group of ≥31 years. Highest achieved level of education was defined into three categories: low level of education (medium- level secondary education or below), medium level of education (higher-level secondary education or vocational education) and high level of education (diploma level or university education). Ethnic origin was defined according to the definition used by Statistics Netherlands, and categorized into Dutch, other western origin, and non-western origin [25]. Statistics Netherlands defines someone to be of non-Dutch origin if at least one of the parents was not born in the Netherlands. In case the parents were born in two different foreign countries, the mother’s country of birth prevailed [25]. Urbanisation level was based on the postal code of the address of the pregnant woman or her partner, stratified according to “area address density” (AAD), and dichotomized into <2499 addresses/km^2^ and >2500 addresses/km^2^[26]. The number of pregnancies women experienced was dichotomized into first and multiple pregnancies. In addition, pregnant women and partners were asked whether they had heard about chlamydia before they participated in the study giving three answer options: “I had heard of chlamydia and knew it was an STI”, “I had heard of chlamydia, but did not know it was an STI”; or “I had never heard of chlamydia before”. Regarding knowledge of chlamydia, twelve questions covered pregnant women’ and partners’ knowledge about the infection. We asked them to indicate which general statements and transmission routes of the infection were “true” “false” or they “did not know”. We presented pregnant women and partners with a list of six general statements of which one was false, and with a list of six transmission routes of the infection, of which three were true and three were false. Knowledge scores were calculated from the knowledge questions and each correct answer was given a value of +1, and an incorrect answer or the ‘don’t know’ option a value of 0. Therefore the overall knowledge sum score could vary between 0 and 12. Regarding the attitudes towards testing, we asked pregnant women and partners whether they agreed with one of five statements about their attitudes towards testing for chlamydia in pregnant women: 1) all women should be tested; 2) only women at increased risk should be tested; 3) only women who want to be tested should be tested; 4) testing during pregnancy is not necessary; and 5) I have no opinion about whether or not pregnant women should be tested. In addition, we asked pregnant women and partners whether or not they thought partners of pregnant women should also be tested for chlamydia during antenatal care if the pregnant woman was tested. Finally, we asked pregnant women and partners about their experiences for being offered a chlamydia test during antenatal care by their midwife. Pregnant women and their partners were asked if they felt satisfied, surprised, stigmatized, ashamed, and whether the test offer had an emotional impact on them. These answers were recorded on a five point Likert scale, graded from 1: “strongly agree” to 5: “strongly disagree”. The statements were dichotomized into two categories: (strongly) agreeing (Likert scale 1–2) with the statement versus neutral or (strongly) disagreeing (Likert scale 3–5) with the statement.

### Chlamydia trachomatis detection

To detect *C. trachomatis* infection, DNA was isolated from the vaginal swab or urine specimen by the High Pure PCR Template Preparation Kit (Roche), and processed using the new CE-IVD certified PRESTO^-PLUS^ test (Goffin Molecular Diagnostics, Houten, the Netherlands). Pregnant women and their partners received the test result by mail. Those who tested positive for chlamydia were advised to contact their general practitioner for treatment. Midwives received the test results of the pregnant women, but not of the partners. In the current antenatal care system the partner is not considered as a midwife’s client. Therefore, midwives did not receive the partners’ test results.

Data from the informed consent forms and the questionnaires were linked with chlamydia test results using anonymized identification numbers.

### Analysis

We calculated frequency distributions for questionnaire items on the separate knowledge questions and the knowledge score, attitudes towards chlamydia testing in antenatal care and experiences for being offered a chlamydia test.

We used the Mann–Whitney U test and Kruskal-Wallis test for differences in knowledge scores between subgroups of pregnant women and their partners based on their characteristics. We used these non-parametric tests because the knowledge score was not normally distributed. In addition we used *X*^*2*^-test statistics to test for differences in knowledge questions between subgroups of pregnant women and partners, and for differences between pregnant women and partners in the experiences of being offered a test. For all analyses we used SPSS 20.0 (SPSS inc., Chicago, IL).

## Results

In total 485 pregnant women from 22 primary midwifery care practices participated in this study. Of them, 102 pregnant women were excluded from analysis: five did not have a unique participation code, and 97 did not return the questionnaire. After exclusion, 383 pregnant women remained in the study, of whom 286 partners participated. Four partners were excluded from the study because they did not return the questionnaire, resulting in 282 partners included for analyses.

### Characteristics of the participants

The median age of the pregnant women was 27 years, range 18 to 30 years. The median age of the 282 partners was 30 years, range 18 to 49 years. Vaginal swabs and urine samples were available from 627 participants (94.3%), of which 14 (2.2%) tested positive for chlamydia; seven women (1.9%) and seven partners (2.6%). Two women tested negative while their partner tested positive. More detailed information about background characteristics of the participants is shown in Table 1.

**Table 1:** Characteristics and median knowledge scores of pregnant women and partners

**Subgroups**	**Number**^**a **^**(%)**	Median knowledge score*	**P-value**^**b **^**Differences in knowledge scores per subgroup**
*Chlamydia trachomatis* (n = 627)			.15
Positive negative	14 (2.2)	8.0	
	613 (97.8)	9.0	
Participants (n = 665)			<0.001
Pregnant woman partner	383 (57.6)	9.0	
	282 (42.4)	9.0	
Age group (n *=* 665)			.02
≤ 20 years	30 (4.5)	8.5	
21-25 years	147 (22.1)	9.0	
26-30 years	383 (57.6)	9.0	
≥ 31 years (partners only)	105 (15.8)	9.0	
Ethnic origin (n = 659)			<0.001
Dutch	492 (74.7)	9.0	
Other western origins	55 (8.3)	9.0	
Non-western origins	112 (17.0)	8.0	
Urbanisation (n = 655)			.83
<2499 addresses/km^2^	529 (80.8)	9.0	
>2500 addresses/km^2^	126 (19.2)	9.0	
Educational level (n = 662)			<0.001
Low	119 (18.0)	8.0	
Intermediate	255 (38.5)	9.0	
High	288 (43.5)	9.0	
Marital status (n = 381)			.62
Single	10 (2.6)	9.5	
Partner, but not living together	35 (9.2)	9.0	
Married or living together	336 (88.2)	9.5	
Pregnancy planned (n = 383)			.21
No	100 (26.1)	9.0	
Yes	283 (73.9)	10.0	
First pregnancy (n = 376)			.09
Yes	217 (57.7)	10.0	
No	159 (42.3)	9.0	
History of STI (n = 654)			<0.001
No	225 (34.4)	9.0	
Yes	87 (13.3)	10.0	
Never been tested	342 (52.3)	9.0	

*Minimum possible score = 0; Maximum possible score = 12.

^a^Denominator varies due to missing variables (between 0 and 11 missing per item) or data is only available for pregnant women.

^b^Mann–Whitney U test and Kruskal-Wallis test.

### Knowledge of Chlamydia trachomatis infection

In total, 616 (92.8%) pregnant women and their partners had heard about chlamydia before they participated in this study and knew that the infection was an STI; 5 (0.8%) of them had heard of chlamydia but did not know the infection was an STI, and 43 (6.5%) pregnant women and partners had never heard of chlamydia before they participated in this study.

Of pregnant women and partners, 81 (12.3%) answered all twelve knowledge questions correctly. The overall median knowledge score was 9 out of a maximum possible score of 12 (range: 0 to 12); and 17 (2.6%) of them scored 0. Table 1 shows the median knowledge scores per demographic subgroup. Significantly higher knowledge scores were found among the following subgroups: pregnant women, age ≥ 21 years, Dutch and other western origins, high educational level and a history of STIs. The correct answers on the knowledge questions are shown in Table 2. The median knowledge scores for pregnant women was 9 out of a possible score of 12 (25th percentile 8, 75th percentile 11); for partners the median knowledge score was 9 out of a possible 12 (25th percentile 7, 75th percentile 10). In general, pregnant women had more knowledge on both the true and false statements than partners. Significant differences between pregnant women and partners in correct answers were found for the statement that chlamydia can be cured by medicines (89.2% of pregnant women versus 78.6% of partners); that you can have a chlamydia infection more than once (67.2% of pregnant women versus 58.0% of partners); and that chlamydia can cause infertility (73.5% of pregnant women versus 64.4% of partners). In addition, pregnant women indicated significantly more often correctly “no” to the statement that you always have symptoms when you are infected (85.3% of pregnant women versus 71.5% of partners). Pregnant women and partners were aware that chlamydia infections can be transmitted by genital sexual contact with an infected person.

**Table 2:** **Knowledge concerning *Chlamydia trachomatis *** **infection**

**Knowledge statements**	**Pregnant women (n = 383) N (%)**	**Partners (n = 282) N (%)**	**P-value**^**a**^
*General questions about Chlamydia trachomatis*			
*True answers*			
Can you infect people without knowing it?	336 (88.2)	236 (84.0)	.15
Can chlamydia be cured with medicines?	340 (89.2)	221 (78.6)	<0.001
Can you have chlamydia more than once?	256 (67.2)	163 (58.0)	.02
Can chlamydia cause infertility?	280 (73.5)	181 (64.4)	.02
Is condom use protective against chlamydia?	300 (78.7)	216 (76.9)	.63
*False answers*			
Will you always have symptoms when infected?	325 (85.3)	201 (71.5)	<0.001
*Chlamydia trachomatis can be transmitted by:*			
*True answers*			
Genital sexual contact with an infected person	349 (91.8)	250 (89.0)	.26
Anal sexual contact with an infected person	246 (64.7)	169 (60.4)	.29
Oral sexual contact with an infected person	212 (55.8)	146 (52.1)	.40
*False answers*			
Kissing an infected person on the mouth	290 (76.3)	196 (70.0)	.08
A toilet seat	241 (63.4)	154 (54.8)	.03
Sharing bath towels with an infected person	218 (57.4)	135 (48.0)	.02

^a^Chi square test.

Pregnant women significantly more often correctly indicated that you cannot get infected with chlamydia through toilet seats (63.4) than partners (54.8%). In addition, women indicated significantly more often correctly that one cannot get infected with chlamydia through bath towels (57.4%) than partners (48.0%).

### Attitudes towards testing

According to 347 (54.2%) participating pregnant women and partners, all women should routinely be tested for chlamydia in antenatal care; 85 (13.3%) reported that only women at increased risk should be tested; 160 (25.0%) reported that pregnant women should only be tested if they want to be tested, one persons reported that testing pregnant women for chlamydia was not necessary, and 47 (7.3%) reported that they had no opinion about whether or not pregnant women should be tested for chlamydia in antenatal care. Table 3 shows the differences in attitudes between pregnant women and partners towards testing pregnant women for chlamydia. Compared to the pregnant women, partners were less likely to report that only pregnant women at increased risk should be tested for chlamydia (8.8% partners versus 16.6% pregnant women) and partners were more likely to have no opinion whether or not pregnant women should be tested for chlamydia during pregnancy (10.7% partners versus 4.9% pregnant women). In addition, 512 (78.3%) of the participants indicated that the partners should also be tested for chlamydia during pregnancy if the pregnant women was tested; 48 (7.3%) indicated it was not necessary to test also the partner, and 94 (14.4%) did not have an opinion about whether partners of pregnant women should be tested. Compared to the partners, pregnant women indicated more often that testing partners for chlamydia during pregnancy was not necessary (10.8% of pregnant women versus 2.6% of partners, p-value <0.001). Partners indicated more often than pregnant women that they did not have an opinion about the statement that it is necessary to test partners of pregnant women for chlamydia during antenatal care (10.3% of pregnant women versus 20.1% of partners, p-value 0.001).

**Table 3:** **Attitudes towards *Chlamydia trachomatis *** **testing during antenatal care**

**Attitudes towards chlamydial testing**	**Pregnant women (n = 368) N (%)**	**Partners (n = 272) N (%)**	**P-value**^**a**^
All pregnant women should be tested	206 (56.0)	141 (51.8)	.34
Only pregnant women at increased risk	61 (16.6)	24 (8.8)	.01
Only pregnant woman who want to be tested	83 (22.6)	77 (28.3)	.12
Testing pregnant women is not necessary	0 (0.0)	1 (0.4)	.88
No opinion	18 (4.9)	29 (10.7)	.01

^a^Chi-square test.

### Experiences of being offered a test

The majority of pregnant women (59.2%) and partners (56.2%) felt satisfied with the test offer for chlamydia, and for most pregnant women (70.5%) and partners (69.7%) it had no emotional impact (Table 4). In total, 3.7% of the pregnant women and 1.8% of partners felt stigmatized by having a chlamydia test offered, and 2.7% of the pregnant women and 1.1% of the partners felt ashamed by having a test offered.

**Table 4:** **Experiences of being offered a *Chlamydia trachomatis *** **test during antenatal care**

**Experiences**	**Pregnant women who agree**^**a **^**(n = 376) N (%)**	**Partners who agree**^**a **^**(n = 274) N (%)**
I felt satisfied with the test offer	221 (59.2)	154 (56.2)
I felt surprised by the test offer	57 (15.2)	48 (17.5)
I felt stigmatised by the test offer	7 (1.8)	10 (3.7)
I felt ashamed by the test offer	10 (2.7)	3 (1.1)
The test offer had no emotional impact on me	265 (70.5)	191 (69.7)

^a^Percentage of pregnant women and partners who strongly agree or agree with the statement.

## Discussion

This study shows that pregnant women and their partners think that testing women for chlamydia during antenatal care is acceptable and not stigmatizing.

To our knowledge there are not many studies in industrialized country that tested both pregnant women and partners for chlamydia infection during antenatal care, as well as that we assessed their attitudes towards testing and experiences of being offered a test. We found positive attitudes towards screening. However, it is possible that partners of pregnant women who did not participate in this study were less positive about being tested for chlamydia infection during antenatal care. Some bias may have occurred in this study. We cannot comment on the characteristics or reasons for not participating since the number and reasons for refusal for both pregnant women and their partners were not recorded. Furthermore, midwives may not have asked all eligible pregnant women to participate. Possible explanations may be time constraints or because midwives felt uncomfortable asking pregnant women to participate in a chlamydia study. In our previous study we have shown that midwives are often not comfortable asking pregnant women about their sexual history. Likewise, they may feel uncomfortable inviting women and partners to participate in a chlamydia prevalence study [18]. In addition, 20% of pregnant women did not return the questionnaire. It is possible that the women and partners did not have the commitment to participate, but also that they did not know the answers to the questions and therefore not returned the questionnaire. Furthermore, pregnant women and partners completed the questionnaire at home and may have searched the Internet for correct answers on the knowledge questions. These facts may have led to an overestimation of the knowledge scores and an overoptimistic view on screening for chlamydia in pregnancy and explain the differences with other studies among pregnant women and non-pregnant young women in which lower awareness levels and knowledge scores for chlamydia infection were found [20,27]. In addition, our respondents were higher educated than the general Dutch population. This may also explain why we found lower prevalence rates of chlamydia in pregnant women compared with previous studies.

Unfortunately, we did not investigate the attitudes and experiences of pregnant women and partners after they received their chlamydia test result. A positive test result may influence their future attitudes or experiences [28]. However, studies from Australia showed that chlamydia infected women, both pregnant and non-pregnant, did not differ from uninfected women concerning their attitudes towards testing, and most of them felt relieved and grateful that chlamydia was diagnosed and treated [20,28]. Testing might be acceptable for pregnant women as they could undertake whatever care is necessary to ensure the health of their baby [20].

The majority of the pregnant women and their partners included in this study were aware about chlamydia being an STI, unlike the study among pregnant women in Australia [20]. Again, these results may be an overestimation of the actual level of awareness among pregnant women and their partners, as the correct answer was given as one of the answer options. Our results show differences in knowledge scores between certain subgroups of participants. Lower knowledge scores were found among partners, pregnant women and partners aged 21 years and younger, pregnant women and partners of non-western origin and pregnant women and partners with a low educational level. These findings are indirectly comparable with the differential uptake of chlamydia screening programs in the general population, as these subgroups often have lower participation rates [29]. This is important, as these subgroups are also at higher risk for chlamydia infection [1,8,20].

Our study found that pregnant women and their partners had positive attitudes towards antenatal chlamydia testing. Although one quarter of the pregnant women and partners indicated that pregnant women should only be tested if they want to be tested, the majority indicated that all pregnant women should be tested for chlamydia. This indicates high acceptance of testing for chlamydia during antenatal care. Furthermore, the majority of participants indicated that the partner of a pregnant woman should also be tested for chlamydia infection during antenatal care. These results are comparable with a study from Sweden in which most of the interviewed men showed positive attitudes towards testing for HIV and chlamydia during antenatal care and that this would make them feel more involved in the pregnancy [30]. This may also explain that partners indicated more often than pregnant women that testing the partners for chlamydia during pregnancy was necessary. Partners are often seen as a psychosocial support for the pregnant woman, but the biological health risks of transmitting an STI to the women and their unborn offspring are usually neglected [30]. Testing partners for chlamydia may be important, as a Dutch study among asymptomatic couples showed that at one time-point only half of the partners were infected [31]. Hence, it may be possible that a woman tests negative for chlamydia during the first trimester of pregnancy while her partner has a chlamydia infection, which occurred twice in our study. In that case the pregnant women might get infected by her partner later during pregnancy. Midwives in the Netherlands provide only care to pregnant women and not to their partners. However, women and partners showed positive attitudes towards partner testing during pregnancy, which may offer an opportunity to add this screening to the midwifery scope of practice or to arrange for testing by a general practitioner or an STI clinic.

The majority of pregnant women and partners felt satisfied when they were offered a chlamydia test. Only a small proportion felt stigmatized or ashamed when the midwife offered them a test. For midwives it is necessary to minimize embarrassment by offering clients appropriate information on chlamydia infection. In the Netherlands, target screening for chlamydia is recommended by the Dutch Health Council [17]. However, target screening has the potential to stigmatize people, and midwives may not feel comfortable in asking their clients questions about sexual behaviours [18]. In addition, Dutch midwives usually base their decision to offer pregnant women a chlamydia test on symptoms of the disease [18]. Hence, many cases of chlamydia remain undetected and untreated, as chlamydial infection causes symptoms in only 20% of women [2]. Routine screening of all pregnant women will prevent stigmatization. A study that estimated the cost-effectiveness of chlamydia screening among Dutch women revealed that screening women for chlamydia during pregnancy is cost-effective in the Netherlands [16]. Moreover, pregnant women are often highly motivated to accept chlamydia testing during antenatal care, as they are willing to undertake whatever care is necessary to ensure the health of their offspring [20].

## Conclusion

This study showed that pregnant women and their partners were knowledgeable about chlamydia infection and that testing was highly acceptable and not stigmatizing. These results provide a good basis for introducing a chlamydia screening programme during pregnancy in the Netherlands. Since chlamydia can be easily treated, such program would lower transmission of chlamydia, maternal disease, adverse pregnancy outcomes and neonatal disease.

## Competing interests

The authors declare to have no competing interests.

## Authors’ contributions

MTRP, JM, ERS, FGS and EKH developed the study protocol. MTRP, JM, ERS, GIJGR, SAM, FGS and EKH developed the questionnaire for pregnant women and partners. MTRP collected the data and was responsible of data linkage of the data sources. SAM analysed the *C. trachomatis* samples. MTRP, JM, ERS, GIJGR, SAM, FGS and EKH supported the data analyses. All authors contributed to the editing of the manuscript and have reviewed and approved the final version.

## Pre-publication history

The pre-publication history for this paper can be accessed here:

http://www.biomedcentral.com/1471-2458/14/704/prepub
